# Moving forward in global-change ecology: capitalizing on natural variability

**DOI:** 10.1002/ece3.433

**Published:** 2013-01-10

**Authors:** Inés Ibáñez, Elise S Gornish, Lauren Buckley, Diane M Debinski, Jessica Hellmann, Brian Helmuth, Janneke HilleRisLambers, Andrew M Latimer, Abraham J Miller-Rushing, Maria Uriarte

**Affiliations:** 1 School of Natural Resources and Environment, University of MichiganAnn Arbor, Michigan; 2 Department of Biological Science, Florida State UniversityTallahassee, Florida; 3 Biology Department, University of North CarolinaChapel Hill, North Carolina; 4 Department of Ecology, Evolution and Organismal Biology, Iowa State UniversityAmes, Iowa; 5 Department of Biological Sciences, University of Notre DameNotre Dame, Indiana; 6 Environment and Sustainability Program and Department of Biological Sciences, University of South CarolinaColumbia, South Carolina; 7 Biology Department, University of WashingtonSeattle, Washington; 8 Department of Plant Sciences, University of California, DavisDavis, California; 9 National Park Service, Schoodic Education and Research Center and Acadia National ParkBar Harbor, Maine; 10 Department of Ecology, Evolution and Environmental Biology, Columbia UniversityNew York, New York

**Keywords:** Climate change, environmental gradients, forecasting, range shifts, translocation

## Abstract

Natural resources managers are being asked to follow practices that accommodate for the impact of climate change on the ecosystems they manage, while global-ecosystems modelers aim to forecast future responses under different climate scenarios. However, the lack of scientific knowledge about short-term ecosystem responses to climate change has made it difficult to define set conservation practices or to realistically inform ecosystem models. Until recently, the main goal for ecologists was to study the composition and structure of communities and their implications for ecosystem function, but due to the probable magnitude and irreversibility of climate-change effects (species extinctions and loss of ecosystem function), a shorter term focus on responses of ecosystems to climate change is needed. We highlight several underutilized approaches for studying the ecological consequences of climate change that capitalize on the natural variability of the climate system at different temporal and spatial scales. For example, studying organismal responses to extreme climatic events can inform about the resilience of populations to global warming and contribute to the assessment of local extinctions. Translocation experiments and gene expression are particular useful to quantitate a species' acclimation potential to global warming. And studies along environmental gradients can guide habitat restoration and protection programs by identifying vulnerable species and sites. These approaches identify the processes and mechanisms underlying species acclimation to changing conditions, combine different analytical approaches, and can be used to improve forecasts of the short-term impacts of climate change and thus inform conservation practices and ecosystem models in a meaningful way.

## Introduction

The need to understand and forecast responses of communities and ecosystems to climate change has become increasingly urgent in ecological research (Pressey et al. 2007; Gilman et al. 2010; Pettorelli 2012). As a response, the scientific community has been approaching climate-change research and its impacts on societies through the use of climate scenarios for the next few decades (20–100 years). However, this approach has also placed the issue of climate change and its consequences in a time frame that is far beyond the one in which policy and decision makers most frequently operate (5–10 years). In addition, the spatial scales of climate scenarios that can be established with the best available tools and methods (i.e., regional models) still have a much larger spatial scale than the ones often needed for actual decision-making (i.e., the local level) (Sinclair et al. 2010). The challenge of effectively incorporating the information resulting from climate-change research into decision-making is thus complicated by this “double conflict of scales.”

We propose that one of the most effective ways to resolve this conflict and to predict community responses is to study how communities and ecosystems respond to current and past climate variability. Our goals here are to demonstrate how we can capitalize on natural variability – variability in organisms' performance along spatial and temporal gradients of environmental conditions (Box 1) – studying the mechanisms underlying ecosystems' short- to mid-term (∼5–50 years) responses to climate change. In doing so, we also highlight the use of analytical methods and alternative sources of information to supplement current approaches. These methods optimize the use of available information and can improve the reliability of our predictions by better exploring the range of potential outcomes of ecosystem responses to climate change.

**Box 1. List of key terms, and their definitions, used in this review**

- *Natural variability*: variability in organisms, populations, or species' performance along spatial and/or temporal gradients of environmental conditions.
- *Acclimation potential*: phenotypic organism, population or species' responses that facilitates and optimal level of performance. Mainly referred in the text as short-term (5–50 years) responses to climate change.
- *Adaptation potential*: evolutionary responses to change, implying genetic changes and natural selection. Mainly relevant for long-term responses to climate change (multigenerational dynamics).

Ecological and evolutionary responses will both drive climate-change effects on ecosystems. Ideally, we should evaluate them simultaneously (e.g., Skelly et al. 2007; Urban et al. 2012a), but the approaches and methods required to study each type of response are quite different (Lavergne et al. 2010). And, although micro-evolutionary responses to environmental change (adaptation; Box 1) can take place in the order of decades (Huey et al. 2000; Jump and Penuelas 2005; but see Agrawal et al. 2012), for most organisms, particularly long-live species, their evolutionary rates will be too low to keep up with the pace of climate change (Chown et al. 2010). For these organisms, their main response to environmental change will be ecological, that is, to persist they will have to acclimate (Box 1) or migrate. In this review, we focus on the approaches we believe to be best to evaluate organisms' acclimation potential to climate change, the type of response that will likely drive major ecosystems' changes in this time frame (∼5–50 years). These approaches illustrate creative ways that supplement current research by leveraging information already present in the system of study to predict species' short-term acclimation potential, to global warming.

By taking advantage of the intrinsic genotypic and physiological variation that species exhibit in their responses to climatic variability – temporal at one location or spatial along its distributional range – these approaches provide a robust framework for studying the potential responses of many species to climate change. For example, studies along climatic or edaphic gradients (e.g., tidal zones, elevational and latitudinal gradients, mosaics of soil types), monitoring programs carried out over mid- to long-temporal scales (>5 years), and studies that exploit the geographic variability on species' performances (e.g., translocation and environmental gradients experiments) all capitalize on natural variability gradients. These approaches are very flexible and relatively inexpensive, can be easily replicated across sites, and can be effectively applied to a wide variety of locations and systems making them highly feasible for most researches. Thus, our goal in this review is to encourage global-change researchers to capitalize on natural variability to study species and ecosystems responses to climate change. We do not advocate this as the only path to pursue, but as an approach available to most researchers that is currently underutilized, and that, given its great potential, could greatly advance the field of global-change ecology.

## Prevailing Approaches

### Habitat suitability models

Information gathered from species' responses to past climatic changes and from climate envelope models has been used to predict habitat suitability for many species. Although these reconstructions and modeling outcomes can be useful predictors of long-term responses on a coarse scale, they are limited in their ability to forecast changes in the shorter term for a number of reasons. Their predictions do not explicitly account for species interactions that take place at finer scales, such as competition (Clark et al. 2011; Urban et al. 2012b), herbivory (Trotter et al. 2002), or predation (Harley 2011). These biotic processes are critical to understanding how species may acclimate to regional changes; ignoring them can result in overestimations of suitable habitat (Preston et al. 2008; Gilman et al. 2010). Also, because they are purely correlative, non-mechanistic fits, they cannot extrapolate reliably to non-analogous future climate scenarios (Helmuth et al. 2005).

### Manipulative experiments

Manipulative experiments provide data describing how organisms respond to modified environmental conditions (e.g., soil and air warming experiments and precipitation manipulations). Although these approaches are useful for studying individuals' physiological responses to climate change, they may not represent real conditions or forecasted scenarios (Beier et al. 2012). Moreover, they are limited in their spatial and temporal extent (Leuzinger et al. 2011). Financial and temporal constraints require that experiments be conducted at small spatial scales and for short periods of time, making extrapolation of results to larger areas and longer temporal scales difficult. Also, because they are often embedded in a surrounding control landscape that may still act as a constant source of propagules to the “sink” experimental area, manipulative experiments can fail to evaluate species turnover that might occur as a consequence of climate change, thereby missing potential shifts in magnitude and direction of species interactions under the new environment.

### Physiological studies

The field of macrophysiology (e.g., Gaston et al. 2009) has provided considerable insight into how the physiological performance of organisms drives their limits to abundance, distribution, and reproductive performance. It therefore provides alternative mechanisms for forecasting responses that go beyond existing environmental conditions. These methods have been successfully applied to a number of organisms and can incorporate biotic factors such as predation, competition (Pincebourde et al. 2008), and behavior (Kearney et al. 2011). However, they are also limited by the inclusion of a low number of environmental stressors, inadequately reflecting the complex environment species will be experiencing (Zarnetske et al. 2012). A recent survey (Crain et al. 2008) showed that, under natural field conditions, ecosystems are often highly unpredictable when exposed to multiple stressors, suggesting that, although models based on single factors such as temperature may serve as a useful starting point, they should ultimately incorporate more complex interactions (Paine et al. 1998).

Despite their limitations, these approaches have produced valuable insights. For example, one of the major lessons learned from past reconstructions and habitat suitability models is that climate change will probably have a large and dramatic impact on species distributions. And, manipulative experiments have allowed us to identify the physiological responses of many species to predicted future conditions (Parmesan and Matthews 2006). Still, very little information is available about climate-change effects on biotic interactions (Zarnetske et al. 2012) and, more importantly, about the capacity for short-term acclimation of most organisms to the new environment (Parmesan and Yohe 2003). These shortcomings limit our ability to forecast the full extent of climate change impacts on species and ecosystems, especially at the temporal and spatial scales meaningful for management and conservation.

## Toward more Relevant Levels of Complexity

Here, we identify fruitful and underused avenues that represent exciting complementary directions for research in global-change ecology. These approaches fall into two broad categories: (1) Capitalizing on Natural Variability and (2) Combining Information and Alternative Analytical Approaches. By overcoming key limitations of the methods listed above, these approaches are important complementary methods that will strengthen the fields' overall research program. They address critical but unanswered questions including “What are the short-term responses of populations, species, and ecosystems to climatic variability?” “What is the acclimation capacity of organisms to current climate change?” “What are the key drivers of those responses?” “What are the mechanisms behind species responses to global warming?” And “How much do populations within species differ in their responses?” These are all questions that must be answered in order to generate reliable predictions of future ecosystems' responses to climate change and to develop successful management and conservation practices.

### Capitalizing on natural variability

Forecasting future changes of species and communities in response to climate change requires understanding both the relationship between species performance and the climatic variables likely to change. Here, we briefly describe how studies can take advantage of species past and present responses to spatial and temporal variability in climatic variables to explore future responses to climate change. Specifically, we discuss three approaches that capitalize on this variability, and that in some cases, also incorporate analytical techniques that maximize the use of the information inherent in the data allowing for more realistic predictions (Table 1).

**Table 1:** List of complementary methods proposed, and their main features that capitalize on natural variability to study short-term species' responses to climate change

	Capitalizing on natural variability		
	
Method's features	Mechanistic niche models	Translocation experiments and gene expression	Studies along environmental gradients
Addresses these questions	Acclimation potential	Acclimation potential	Acclimation potential
	Key drivers	Key drivers	Key drivers
	Underlying mechanisms	Variability among populations	
Complements these prevailing approaches	Habitat suitability models	Habitat suitability models	Habitat suitability models
	Physiological studies	Manipulative experiments	Manipulative experiments
Supplemented by these Information and analytical approaches	Hybrid models	Hybrid models	Hybrid models
		Alternative sources of information	Remote sensing data
Strengths	Links environment with organisms' performance and with population demography	Assesses intra-species variability	Takes into account a wide array of driving variables
	Identifies range limits	Identifies range limits	Identifies concrete climatic drivers
			Provides vulnerability assessments
Weaknesses	Requires detailed study of the organisms	Limited number of genes sampled	May required mid- to long- term data
	Not generalizable to other species (or genotypes)	Based on RNA sequencing	It may be difficult to isolate the specific response to climate change
Potential to capitalize on natural variability	Temporal: medium-low	Temporal: low	Temporal: medium-low
	Spatial: medium-high	Spatial: medium-high	Spatial: medium
Feasibility	Medium (may required advance quantitative skills)	Medium (requires genetic lab)	High (although advance modeling will require quantitative skills)

Variability in climate, including the incidence of extreme events, is a useful tool for the evaluation of species' and ecosystems' responses to future climate conditions (Gornish and Miller 2010). Its advantage is that the range of annual conditions experienced in one location spans that of recent near-term forecasts of climate change (∼20–40 years), making extrapolation of future performance feasible. This approach also allows us to discern which climatic drivers influence performance, for example, annual as opposed to seasonal, extreme events, interactions between covariates (e.g., Helmuth et al. 2010), the mean as opposed to changes in variability (e.g., Stachowicz et al. 2002), and the nature of the relationship (e.g., linear as opposed to saturating or quadratic). And with respect to extreme events, changes in environmental means will likely play a smaller role in the evolution of species performance to climate change than will extreme events (Angilletta et al. 2006; Chown and Terblanche 2007).

Comparing the performances of species or populations in locations that differ in climate (space-for-time substitution) can also help forecast ecological impacts of climate change. Habitat suitability models are the simplest of these comparisons, correlating the presence of species to spatial variation in climate and using such relationships to predict future distributions with climate change. More mechanistic data than simply presence–absence (e.g., abundance, reproductive success) can lead to even greater insight into the relationship between climate and species performance (e.g., physiological tolerances (Deutsch et al. 2008); gene expression), and thus the manner in which the changing climate is likely to influence species distributions.

Examining the potential interactions between effects of spatial and temporal climate variability on community and ecosystem dynamics and defining the potential drivers of such change provides a powerful approach for evaluating community dynamics and ecosystems resilience to future climate (Table 1).

#### Mechanistic niche models

Mechanistic niche models explicitly describe the processes by which organismal traits interact with environmental conditions to determine individual energetics and population dynamics (Kearney and Porter 2009; Monahan 2009; Buckley et al. 2010). These models assume a strong relationship between climate factors and distribution limits, and provide a framework for examining the implications of temporal and spatial variability in both the environment and organismal traits (Table 1). For example, work done for the skipper butterfly *Atalopedes campestris* showed that the northward range expansion accelerated when warming occurred faster in winter than in summer (Crozier and Dwyer 2006). In another butterfly study, a model was used to show that extended flight durations in response to recent increases in climate means likely had a stronger population impact than did corresponding decreases in egg viability due to an increased incidence of extreme heat events (Buckley and Kingsolver 2012).

**Figure 1:**
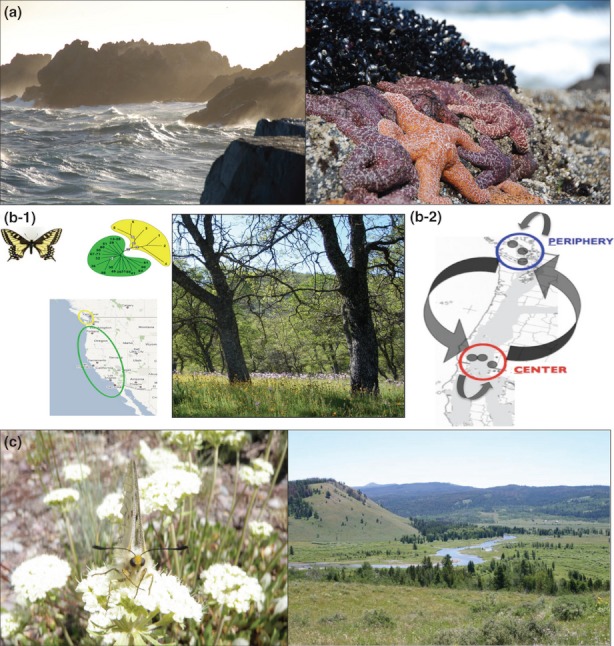
(a) Species performance along the environmental gradient of the intertidal zone can be monitored to assess future outcomes under changing conditions (Helmuth et al. 2005). (b) Translocation experiments and genetic studies can be combined to assess intra-species differential responses to climate change. Zakharov and Hellmann (2008) identified distinct butterfly genotypes (peripheral yellow, core green) in oak savanna ecosystems of coastal North America (1). Pelini et al. (2009) tested the role that local adaptation may play in the species' responses to future climate (2). (c) Debinski et al. (2010) used a hydrological gradient to study differential changes in species composition of meadow communities during drought conditions. *All photographs were taken by the authors.

Together with biophysical models, mechanistic niche models can also translate environmental conditions (e.g., air or water temperature, radiation, and wind speed) into the potential body temperature of organisms, allowing us to link the physical environment with a population's demographic data. Such integration enables us to investigate the consequences of a varying environment on organisms. For example, Helmuth et al. (2005) used biophysical models to link the spatially and temporally varying conditions of the intertidal zone with organisms' body temperatures and demographic data, and then assess the impact of future climatic changes (Fig. 1a).

Mechanistic approaches can also be applied to investigate the range of implications of geographic variation in phenotypes. A study of fence lizards found that population-specific morphological and life history traits corresponded to differences in potential ranges (Buckley 2008). The population-specific traits also led to predictions of individualistic responses to climate change, which have frequently been observed in response to past climate change (Williams and Jackson 2007). Population-specific thermal performance curves were found to have implications for both current and potential future distributions of monkey-flowers (Angert et al. 2011). Likewise, variation in the temperature dependence of locomotive performance may be influencing the range expansion of cane toads in Australia (Kolbe et al. 2010). This approach is also applicable to understanding the implications of trait evolution in response to climate change. Indeed, a biophysical model incorporating evolution demonstrated that evolutionary changes in egg desiccation have the potential to facilitate range expansions of dengue mosquitoes in response to climate change (Kearney et al. 2009a). These important insights achieved through mechanistic niche modeling are a key to short-term forecast of species responses to climate change and could not have been achieved by other means. In addition, as the underlying mechanisms are being identified, results from mechanistic niche models can easily be related to the fitness of the studied organism, and thus indicate potential long-term, evolutionary responses, to climate change (Kearney et al. 2009a; Lavergne et al. 2010).

#### Translocation experiments and gene expression

Most traditional approaches used by ecologists to make projections under climate change assume that individual responses are consistently distributed and uniformly genetically constrained across the range of the species (i.e., species identity is the only factor influencing response). However, common-garden and translocation experiments – where individuals are transplanted or moved outside their site of origin – show that populations differ in important ways across a species' range (e.g., Oleksyn et al. 1998). And, where population differences are pronounced, considering a species' response as a whole is not sufficient.

New translocation studies (e.g., Rutter and Fenster 2007; Pelini et al. 2009) that emphasize climatic factors have attempted to remedy the absence of population differentiation from climate-change research (Table 1). In these experiments, populations from key locations within a species' range (e.g., periphery and center) can be compared under both historical and future climates by being located in areas with a different climate. For example, Pelini et al. (2009) carried out a translocation experiment to assess changes in survivorship of two butterfly species and discovered phenotypic differences within their ranges (Fig. 1b); and Zakharov and Hellmann (2008), working in the same system, identified distinct butterfly genotypes between the peripheral and core populations (Fig. 1b). Such experiments tend to exploit environmental gradients to make relatively simple climate comparisons on differential gene expression among populations of a species, and its potential role on species performance under climate change. And, as not all populations may maintain sufficient genetic variation to respond to climate change (Hoffmann et al. 2003), these studies can also evaluate the effect of different levels of genetic variation on a species short-term acclimation potential, and in the long-term adaptation potential, to environmental change.

Translocation experiments also play a role in testing the factors that determine a species' range limit. Crozier (2004) and Marsico and Hellmann (2009), for example, placed individuals outside of their range to determine which factors set the poleward range boundary. Crozier (2004) found evidence for temperature limitation, suggesting that climate change could drive range expansion, but Marsico and Hellmann (2009) found dispersal limitation to be a likely range-limiting factor, suggesting that higher temperatures are unlikely to cause a rapid range shift. This information is crucial in conservation planning, as it allows the assessment of specific populations' dynamics as well as the whole species'.

#### Studies along environmental gradients

We can capitalize on temporal and spatial environmental gradients to evaluate species performance under a wide range of abiotic and biotic conditions (Ibáñez et al. 2007). Although the classic perspectives on species distributional changes are those of higher latitudes and elevation shifts with warming, the real-world manifestation of such patterns is more complex (Helmuth et al. 2002). Mid- to long-term (>5 years) monitoring along environmental gradients can permit estimation of true shifts in the community in response to changing conditions (Table 1). For example, tracking changes along hydrological gradients in terrestrial systems allows classification of habitats and their associated species from xeric to hydric (Debinski et al. 2006; Fig. 1c). Changes in species distribution, abundance, and performance along gradients facilitate a better assessment of species- and habitat-based vulnerabilities within the ecosystem (e.g., Ibáñez et al. 2008; Debinski et al. 2010). In addition, this approach permits direct assessment of the effects of species interactions in organisms' response to climate change, providing crucial information to evaluate effects of changes in species interactions when both acclimation (from long-live organisms) and adaptation (from short-live species) responses may take place simultaneously (Lau and Lennon 2012).

Working along environmental gradients is the most feasible approach for a majority of global-change researchers. Still, it presents challenges. First, such approaches can require mid- to long-term datasets collected over intensive and extensive temporal and spatial extents (Bolker 2009). Second, integration of responses across different studies is most effective if ontogenetic stages and spatial and temporal scales are similar. Finally, disentangling the relative contributions of multiple covariates, including climate, that jointly influence individual performance is complex (Bolker 2009). In many cases, these challenges can be overcome by multi-investigator collaborations intended to ensure uniformity of field methods (e.g., Stokstad 2011) and/or by use of alternative analytical approaches. Data collected along environmental gradients can be analyzed by means of hierarchical or multilevel models that link scales (individual organisms, sites, landscapes, and regions) and make inferences about species performance at each scale and as a function of the many biotic and abiotic factors expected to affect these processes (Clark 2005; Latimer et al. 2006). These models are highly flexible and adaptable to other systems and can readily incorporate new data as they become available. Hierarchical approaches can also facilitate integration of experimental and observational data with process models that encapsulate our understanding of ecological systems (Ogle and Barber 2008). The statistical characterization of the changes observed in forcing variables (e.g., climate, land use) during the last decades can then be used to propose a range of plausible scenarios of species' and/or ecosystems' short-term responses to change. This information can then be directly used by land managers to assess the local risk of species extinction, and consequentially, to guide habitat restoration and/or protection programs.

In spite of their challenges, approaches that capitalize in natural variability can still complement traditional methods (Table 1). These approaches integrate more biologically reasonable factors driving the interaction between climate change and species' response into models, and can provide highly informed predictions of local short- and mid-term responses to climate change, consequentially helping to assign research, management, and conservation priorities.

### Combining information and alternative analytical approaches

Integrating information from different sources, for example, individual case studies, multiple regions, ecotypes, and synthesizing disparate sources, such as remote sensing data, field observations, and historical records, can produce more robust predictions than extrapolations from single locations or systems. Here, we briefly describe several techniques that can be effectively used for predicting species, community, and population responses to change by means of widely available data and methods.

#### Combining remote sensing data with species distributional ranges and individual-based information

Remotely sensed data, including satellite imagery, aerial photographs, and spectroradiometer data, can provide information that simultaneously quantitates temporal and spatial variation in communities, ecosystems, and forcing factors. This type of data can highlight how the type, abundance and productivity of organisms are distributed across space and time in a way that is infeasible with traditional “single point” observational and experimental approaches. This approach has been used to quantitate changes in vegetation (e.g., Chambers et al. 2007) and to test for phenological changes over time, such as the date of snowmelt or vegetation green-up and senescence (e.g., Zhang et al. 2003). Remotely sensed data can also be used to quantitate inter-annual variability in these metrics as well as temporal trajectories related to climate change. Species-distribution data can be linked with landscape data to quantitate responses at the species and community levels (Latimer et al. 2006; Debinski et al. 2006, 2010; Ibáñez et al. 2009), and long-term gradient-based research projects can be coupled with repeated surveys for assessment of changes over time (e.g., Grace et al. 2011). Such combination of data sources allows for relatively accurate assessment of species/community responses and their resilience to environmental variability at a scale that could not be captured with a less interdisciplinary approach.

Although predicting shifts in species distributional ranges has been the core of global-change ecology, recent reviews have emphasized the importance of predicting ecosystem responses unrelated to changes in range boundaries. Mumby et al. (2011) point out that the ecosystem services provided by systems such as coral reefs can decline significantly well in advance of changes in range boundaries. Similarly, other studies have documented changes in abundance (Jarema et al. 2009), reproductive rates (Beukema et al. 2009), and recruitment (Ibáñez et al. 2007) well within species range boundaries. Such patterns suggest that we need approaches that aim to understand how global climate change will affect species' physiological performances (Monaco and Helmuth 2011), and how ecological and evolutionary responses may be constrained by species interactions (Price and Kirkpatrick 2009; Clark et al. 2011).

The merge of detailed weather data and individuals' performances is revealing that considering spatial and temporal variability in both the environment and organismal responses may be central to forecasting climate change impacts. Mislan and Wethey (2011) combined gridded meteorological data to predict patterns of mortality over a geographic gradient by comparing outputs from a biophysical heat budget model to measurements of lethal temperature limits of an intertidal mussel. Kearney et al. (2009b) and Sará et al. (2011) combined biophysical heat budget models with dynamic energy budget models to predict changes in growth and reproductive output of intertidal mussels, using weather station data as inputs. Kearney et al. (2011) used similar approaches to explore the impacts of changes in climate over a 30-year time scale on the population dynamics of lizards at multiple sites within the United States. Using long-term records of climatological data, they explored the importance of using fine-scale (daily) weather data, and showed that decadal trends emerged only when using these finer scale data (as opposed to monthly data). They, moreover, showed the overriding effects of behavior and habitat quality (in this case, burrowing depth) on the sensitivity of the organism to changes in climate, suggesting that animals in good quality habitat could avoid many of the negative impacts of observed climate change.

Responses to climatic extremes can also provide valuable information with respect to the climatic limits and resilience of organisms or communities (Zimmermann et al. 2009). Particularly, at the edges of their distributional ranges, species' survival (trailing edge) and fecundity (advancing edge) can be highly dependent on the occurrence of extreme climatic conditions (Honnay et al. 2002; Lenoir et al. 2008). Thus, individual or community performance during an extreme climatic event can reveal critical information to assess overall responses to climate change.

#### Hybrid models

Environmental niches are most frequently estimated by means of correlative models based on averaged weather conditions. Recently, correlative (niche) and mechanistic approaches have emerged that consider biological and environmental variability when defining an organism's niche (Morin and Lechowicz 2008; Brook et al. 2009). The result, hybrid models allow for the incorporation of spatial and temporal variability in niche models. Including the output of mechanistic models in correlative models provides a means of accounting for spatial and temporal variability (Gallien et al. 2010; Buckley et al. 2011) resulting on more realistic information about a species' potential to acclimate to climate change.

Limitations on number of environmental layers generally prohibit including temporal variability in niche models, but some niche models have incorporated paleoclimatic stability to reveal the importance of past climate changes to current diversity patterns (Araujo et al. 2008). One straightforward but informative approach is to divide localities by phylogeographic lineages. This addresses whether lineages have diverged in their climatic niche and whether this divergence may be important to forecasting responses to climate change (Rissler and Apodaca 2007). Integrating phylogeographic data and niche modeling with paleoclimatic reconstructions can provide insight into species' responses to past climatic shifts and identify refugial populations (Hugall et al. 2002). Other approaches have incorporated aspects of an organism's physiological performance by combining biophysical models with energetic models (Kearney et al. 2011). Additionally, hybrid models combine the advantages of large-scale correlations with process-based mechanisms, making them an optimal tool to forecast species future responses to climate change across scales.

#### Alternative sources of information and coordinating data-collection efforts

Monetary and time constraints can make the collection of data that adequately capture species' responses to natural climate variability via single experiments an infeasible undertaking. The collection of time series data or coordinated data collections can address limitations commonly associated with individual experiments. Pollen cores, ice cores, long-term weather station data, museum specimens, and historical photographic records can all be used to quantitate both spatial and temporal variation in species performances and distributions (e.g., Miller-Rushing and Primack 2008). Museum data, which are becoming increasingly accessible through online databases, can be used to detect species range shifts relative to elevation and latitude over time (e.g., Kerr et al. 2007). Long- to mid-term records of plant and animal phenology have been combined with weather station data to investigate phenological changes in the last few decades of global warming (e.g., Menzel et al. 2006; Ibáñez et al. 2010). And, long-term pollen records have been extensively used not only to reconstruct past vegetation patterns but also to predict ecological responses to future climate change (Jackson et al. 2009). The strength of these data, however, is most apparent when collaborative networks combine them into a cohesive database. Further efforts at coordinating data collection (e.g., by the USA National Phenological Network) and compiling results (e.g., by the National Ecological Observatory Network) will greatly contribute in the generation of the temporally and spatially extensive data advocated above.

## Conclusions

The lack of scientific knowledge about short-term ecosystems' responses to climate change makes generating predictions of future ecosystems and defining effective management practices difficult. To obtain reliable forecasts of the impacts of climate change on ecosystems, we must consider species-specific responses to changing climates, shifting landscapes, variation in local conditions, and interactions among species – the variables that determine the complex environment species will be encountering in the next few decades. To generate such predictions, we must pursue rigorous assessments of global-change impacts on systems of interest that can be realistically translated into management plans and predictive models, especially those focusing on mitigation of global-change impacts. To achieve this goal**,** we can capitalize on the natural variability associated with environmental gradients and pursue techniques that combine different analytical approaches and sources of information.

Observational and experimental work along natural environmental gradients can reveal a system's potential response to varying climatic conditions, and can do so in situ*,* that is, under the array of variables and drivers of change that interact with climate to shape organismal and community responses. These results, coupled with non-traditional analytical techniques, can allow the exploration of the range of potential outcomes beyond what can be detected with traditional approaches. Thus, our message to global-change ecologists is to capitalize on and profit from the natural variability inherent in their systems of study. No single experiment or modeling technique can answer all our questions or inform all our actions; instead, the combination of multiple approaches will be the key to understanding climate-change impacts on populations, communities, and ecosystems. Studying species and ecosystems responses to variable conditions will be an important step toward those objectives.
